# Are Myeloproliferative Neoplasms‐Unclassifiable Really Unclassifiable?

**DOI:** 10.1002/jha2.70284

**Published:** 2026-04-08

**Authors:** Giovanni Barosi, Vittorio Rosti, Robert P. Gale

**Affiliations:** ^1^ Center for the Study of Myelofibrosis Fondazione Istituto di Ricovero e Cura a Carattere Scientifico (IRCCS) Policlinico San Matteo Pavia Italy; ^2^ Centre for Haematology Department of Immunology and Inflammation Imperial College of Science, Technology and Medicine London UK

**Keywords:** bone marrow, ICC, myeloproliferative neoplasm, myeloproliferative neoplasm‐unclassifiable, WHO

1

The World Health Organization (WHO) and the International Consensus Classification (ICC) classify BCR::ABL‐negative classical myeloproliferative neoplasms (MPNs) into three major entities: essential thrombocythaemia (ET), polycythaemia vera (PV) and primary myelofibrosis (PMF) [1, 2]. Both classifications recognize a residual group designated as MPN‐unclassifiable (MPN‐U) by WHO and MPN‐not otherwise specified (NOS) by ICC. These designations are intended to accommodate cases that do not meet all the diagnostic criteria for established MPN subtypes. This heterogeneous category includes early or advanced disease stages, phenotypic overlaps among MPN subtypes, and presentations confounded by concomitant inflammatory or neoplastic conditions. This residual category is diagnostically ambiguous and conceptually unsatisfactory, and several groups have attempted to refine the boundaries.

Abdelaziz et al. recently interrogated data from 30 subjects diagnosed with MPN‐U, identifying three salient features. First, mean diagnostic haemoglobin, leukocyte and platelet values were within normal limits, indicating that a substantial proportion of diagnoses were made despite unremarkable peripheral blood counts. Second, 43% of cases were identified following a thrombotic event, predominantly venous thrombosis in atypical anatomical sites and with several individuals experiencing recurrent thrombosis, including episodes occurring under anticoagulation. Third, the cohort exhibited a favourable prognosis: after a median follow‐up of 2.8 years (range 0.3–18.5), 15‐year survival was 70% and no cases progressed to overt myelofibrosis [3].

This phenotype closely mirrors a subset of individuals we have previously described and proposed as a distinct clinical variant, ‘clonal megakaryocyte dysplasia with normal blood values’ (CMD‐NBV). The conceptual roots of CMD‐NBV trace back to our 1991 description of an ‘atypical myeloproliferative disorder with high thrombotic risk and low disease progression’ [4]. In 2022, we reported 15 unclassified MPN cases sharing distinctive diagnostic and prognostic features suggestive of a unified entity [5]. More recently, we expanded this series to 30 cases, emphasizing hallmark features like those that now appear replicated in the Abdelaziz cohort [6]. In our series, 70% of CMD‐NBV diagnoses were context‐driven, typically arising during evaluation of incidental or symptomatic venous or arterial thrombosis. These individuals demonstrated a high thrombotic burden, particularly in the splanchnic venous system, with major thrombotic events occurring at a rate of 6.6 per 100 subject‐years. At a median follow‐up of 9.1 years, all but one subject was asymptomatic with persistently normal haematological parameters.

Our delineation of CMD‐NBV within the broader MPN taxonomy rests on diagnostic criteria designed to distinguish this variant from MPN‐U and MPN‐NOS. Consistent with the multiparametric diagnostic philosophy endorsed by the WHO and ICC, CMD‐NBV is defined by a characteristic histopathological pattern of megakaryocytic hyperplasia with marked dysplasia, occurring in the setting of normal or near‐normal clinical and laboratory findings (Table 1).

**TABLE 1:** The proposed diagnostic criteria of clonal megakaryocyte dysplasia with normal blood values (CMD‐NBV).

Subjects should be included in this category when all these criteria are met:
(1) Bone marrow fibrosis Grade 0–1; (2) Bone marrow megakaryocyte hyperplasia and dysplasia as described for primary myelofibrosis (myelofibrosis‐type megakaryocyte dysplasia)^a^; (3) Failure to meet the clinical‐haematological WHO criteria for polycythaemia vera, essential thrombocythaemia. Accordingly, subjects should not have a blood platelet concentration > 450 × 10E + 9/L, or haemoglobin > 165 g/L in men and > 160 g/L in women; (4) None of the minor diagnostic criteria of pre‐fibrotic myelofibrosis, that is, leukocytosis ≥ 11 × 10E + 9/L, increased serum LDH level, anaemia not attributed to a comorbid condition, or palpable splenomegaly.

^a^ This criterion included megakaryocyte endosteal dislocation, loose and dense clustering, nuclear dense lobulation, cloudy and bare denuded nuclei, and nuclear cytoplasmic abnormalities consistent with maturation defects. Abnormalities in the other haematopoietic cell lineages do not enter among the diagnostic criteria.

To explore whether a proportion of MPN‐U cases may in fact represent CMD‐NBV, we conducted a review of published MPN‐U case series. Searches of PubMed, Web of Science and Embase, supplemented by manual reference screening, were restricted to English‐language studies published over the past 30 years. Because bone marrow histology is central to CMD‐NBV diagnosis, studies without biopsy data were excluded, as were conference abstracts, expert opinions and consensus statements. Four eligible case series were identified (Table 2) [7, 8, 9, 10, 11]. Two were single‐centre studies, one involved two international centres and one did not specify its setting. Only one study explicitly reported consecutive case ascertainment. Enrolment periods spanned 8–25 years. Sample sizes ranged from 5 to 94 subjects.

**TABLE 2:** Studies and subjects with MPN‐unclassifiable (MPN‐U) analysed in this review.

First author, year of publication, country of subjects’ origin and (# of reference)	Type of study and subjects’ enrolment	Criteria for subjects’ inclusion in the study	No. of cases with MPN‐U diagnosis	No. of cases with an early stage MPN‐U	No. of cases with bone marrow histology potentially meeting the criteria for CMD‐NBV	Information on laboratory covariates addressing towards the diagnosis of CMD‐NBV	Diagnostic conclusions
Gianelli/Iurlo, 2017, Italy/USA, [7, 8]	*Type of study*: observational retrospective; *Subjects’ source*: two reference centres; *Subjects’ selection method: c*onsecutive patients; *Period of subjects’ inclusion*: 2007–2015	Subjects were diagnosed with MPN‐U according to the 2008 WHO classification, then revised according to the WHO 2016 criteria	71	21/71 (29.6%) had bone marrow fibrosis Grade 0 or 1	3/21 (14.3%) had myelofibrosis‐like bone marrow histology	The 3 subjects with early‐stage bone marrow fibrosis and myelofibrosis‐like histology did not have the required clinical‐haematological criteria for CMD‐NBV	No case diagnosed with MPN‐U had the full set of diagnostic criteria for CMD‐NBV
Yun, 2020, Korea, [9]	*Type of study*: observational retrospective; *Subjects’ source*: NR; *Subjects’ selection method*: NR; *Period of subjects’ inclusion*: NR	Subjects were diagnosed with MPN subtypes according to the 2008 WHO classification	5	2/5 (40%) expressed mild bone marrow fibrosis	2/2 (100%)	Both cases had platelet count > 450 × 10^9^/L	No case diagnosed with MPN‐U had the full set of diagnostic criteria for CMD‐NBV
Deschamps, 2021, UK, [10]	*Type of study*: Observation, retrospective; *Subjects’ source*: NR; *Subjects’ selection method*: NR; *Period of subjects’ inclusion*: 1994–2019	Subjects diagnosed with MPN subtypes according to the 2016 WHO classification	82	62/82 (75.6%) had bone marrow fibrosis Grade 0 or 1	47/47 (100%) of subjects with early‐stage bone marrow fibrosis had megakaryocyte hyperplasia: 39/47 (83%) had clustered megakaryopoiesis	78% of early myelofibrosis had thrombocytosis	22% of subjects with early‐stage bone marrow disease are potential candidates for the CMD‐NBV diagnosis
Crane, 2024, USA [11]	*Type of study*: Observational retrospective multicentre (7 institutions); *Subjects’ source*: NR; *Subjects’ selection method*: NR; *Period of subjects’ inclusion*: 2000–2017	Diagnosis of MPN‐U according to the 2016 WHO criteria	94	64/94 (68%) were defined as early‐stage disorder	73% with early‐stage disease had megakaryocyte hyperplasia; 23% had megakaryocyte dysplasia and atypia.	58.7% had leukocyte > 11 × 10^9^/L; 50.8% had platelet count > 450 × 10^9^/L	Less than 23% of early‐stage bone marrow disease are potential candidates for CMD‐NBV

Abbreviations: CBD‐NBV, clonal megakaryocyte dysplasia with normal blood values; WHO, World Health Organization; NR, not reported; MPN‐U, myeloproliferative neoplasm‐unclassifiable

Among 252 reported MPN‐U cases, 149 of whom (59%) exhibited bone marrow fibrosis Grade 0–1, placing them within the spectrum of potential CMD‐NBV. The proportion of cases with Grade 0–1 fibrosis accompanied by either a myelofibrosis‐like megakaryocytic histology or megakaryocyte hyperplasia with dysplasia ranged from 4.2% to 100% across studies, with a mean of 41.8% (*n* = 52). However, the lack of data on spleen size and plasma LDH concentration precludes a definitive CMD‐NBV diagnosis. These data suggest that a subset of cases currently labelled as MPN‐U may be reclassified as CMD‐NBV, although this inference can only be made with low confidence due to incomplete reporting of diagnostic criteria.

Diagnosing CMD‐NBV is inherently challenging because it requires recognizing dysplastic megakaryocytic features like those seen in PMF. This reliance marks a paradigm shift in MPN diagnostics where, without robust quantitative criteria, diagnosis relies on pattern recognition entailing a nuanced evaluation of the spatial and quantitative interplay among myeloid, erythroid and megakaryocytic lineages, as well as scrutiny of megakaryocyte histology abnormalities. These subtle histological features underscore the need for more refined criteria. In alignment with the recommendations of Abdelaziz et al. for MPN‐U, we suggest that rigorous standardization of bone marrow histopathology is needed to accurately diagnose CMD‐NBV.

Given that bone marrow assessment in MPNs is largely qualitative and subjective, we advocate integrating more objective techniques. Computational approaches able to systematically capture key histological attributes of megakaryocytes have demonstrated associations with specific MPN subtypes [12, 13]. This strategy holds promise for advancing a more precise and reproducible definition of the megakaryocyte features that define the heterogeneous landscape of early‐stage MPNs.

In conclusion, we suggest that many people currently diagnosed with MPN‐U or MPN‐NOS may actually have CMD‐NBV. The high thrombotic risk and favourable prognosis of CMD‐NBV have important clinical implications.

## Author Contributions

G.B. conceptualized the study and did the literature review. G.B., R.P.G. and V.R. prepared the typescript.

## Funding

The authors have nothing to report.

## Ethics Statement

The authors have nothing to report.

## Conflicts of Interest

Robert P. Gale is a consultant to BeiGene Ltd., Fusion Pharma LLC, LaJolla NanoMedical Inc., Mingsight Parmaceuticals Inc., Kite Pharma and CStone Pharmaceuticals; Advisor to Antegene Biotech LLC, Medical Director and FFF Enterprises Inc.; Partner in AZACA Inc.; Board of Directors, RakFond Foundation for Cancer Research Support; Scientific Advisory Board, StemRad Ltd. ICC. The other authors declare no conflicts of interest.

## Data Availability

No original data are reported in this manuscript
